# NeuroProtect, a Candidate Formula From Traditional Chinese Medicine, Attenuates Amyloid-*β* and Restores Synaptic Structures in APP/PS1 Transgenic Mice

**DOI:** 10.3389/fphar.2022.850175

**Published:** 2022-05-02

**Authors:** Yan Tan, Xu Wang, Jiani Zhang, Huawei Zhang, Haiyan Li, Tiantian Peng, Weihang Chen, Peng Wei, Zhaoheng Liu, Fang He, Jiao Li, Haimin Ding, Na Li, Zhaoyang Wang, Zhenqiang Zhang, Qian Hua

**Affiliations:** ^1^ School of Life Sciences, Beijing University of Chinese Medicine, Beijing, China; ^2^ School of Traditional Chinese Medicine, Beijing University of Chinese Medicine, Beijing, China; ^3^ School of Acupuncture-Moxibustion and Tuina, Beijing University of Chinese Medicine, Beijing, China; ^4^ Xi’an Satellite Control Center, Xi’an, China; ^5^ Academy of Chinese Medical Sciences, Henan University of Chinese Medicine, Zhengzhou, China

**Keywords:** A*β*, Alzheimer’s disease, geniposide, *Panax notoginseng* saponins (PNS), synaptic structure

## Abstract

**Background:** Alzheimer’s disease (AD) is the most common cause of dementia. The emerging data suggest that cognitive decline occurred in the setting of A*β* accumulation with synaptic dysfunction, which started to happen at preclinical stages. Then, presymptomatic intervention is more critical to postponing AD processing. Traditional Chinese medicine has a long history of treating and preventing dementia. Findings have shown that the decoction of *Panax notoginseng* and *Gardenia jasminoides* Ellis enhances memory functions in patients with stroke, and their main components, *Panax notoginseng* saponins (PNS) and geniposide (GP), improved memory abilities in experimental AD models. Since herbal medicine has advantages in protection with few side effects, we wish to extend observations of the NeuroProtect (NP) formulation for reducing amyloid-*β* and restoring synaptic structures in APP/PS1 transgenic mice.

**Methods:** APP/PS1 transgenic mice and their wild-type littermates were fed with control, NP, and their components from 4 to 7 months of age. We assessed the synaptic structure by Golgi staining, analyzed the amyloid deposits by Thioflavin-S staining, and measured related protein levels by Western blot or ELISA. We used the Morris water maze and shuttle box test to evaluate cognitive functions.

**Results:** Compared to WT mice, APP/PS1 mice are characterized by the accumulation of amyloid plaques, reducing synaptic structure richness and memory deficits. NP prevents these changes and ameliorates cognitive deficits. These effects may have been due to the contribution of its components by inhibition of insoluble amyloid-*β* deposition and restoration of synaptic structures.

**Conclusion:** These findings reveal a beneficial effect of NP on AD progression under an early intervention strategy and provide a food supplement for AD prevention.

## Graphical Abstract

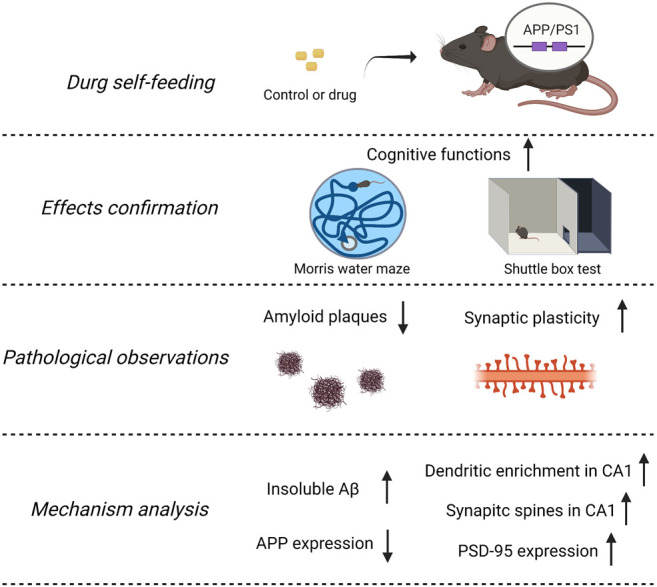

## Introduction

Alzheimer’s disease (AD), the primary common type of dementia in the geriatric population, is a neurodegenerative disorder characterized by progressive memory loss and cognitive decline (Masters, et al., 2006). It refers to the combined presence of amyloid *β* (A*β*), tau, synaptic dysfunction, and neuron loss (Ballard, et al., 2011; Barthélemy, et al., 2020; Panza, et al., 2019; Tu, et al., 2014; van der Kant, et al., 2020). Cerebrospinal fluid (CSF) A*β*42 or PET amyloid imaging is the earliest dynamic (Jack, et al., 2013). Also, synaptic dysfunction is another proximate cause of subtle cognitive impairment in early AD (Qi, et al., 2021). The emerging data suggest that the cognitive decline occurred only in the presence of A*β* accumulation with synaptic dysfunction (Sperling, et al., 2011). In 2018, the Food and Drug Administration (FDA) released a position statement titled “Early Alzheimer’s Disease: Developing Drugs for Treatment, Guidance for Industry”; it highlighted that the efforts were particularly significant to intervene very early in the AD processing (U.S. Department of Health and Services, 2018). Then, identifying and targeting these early markers altogether are the hopes of investing in this field of study (John and Reddy, 2021).

Traditional Chinese medicine (TCM) is an ancient and effective medicinal system extensively oriented from China. TCM is widely used in East Asia to prevent and treat neurological diseases, including stroke and dementia (Bu, et al., 2020; Li, et al., 2021; Liu, et al., 2018). TCM doctors prescribe herbal formulation rather than single substance drug in the clinic because of the complex pathogenies and multi-symptoms of diseases. Our previous study found that the combination of *Panax notoginseng* and *Gardenia jasminoides* Ellis can improve cognitive functions in acute stroke patients (Chinese SFDA: 2004 L01620). To optimize the formula, we found that their main components, *Panax notoginseng* saponins (PNS) and geniposide (GP) (newly named NeuroProtect, NP), can effectively improve the blood supply to cerebral tissues, thus recovering the neurotrophic effects of the cerebral microenvironment (Hua, et al., 2010; Li, et al., 2012). Furthermore, by three AD-like animal models, NP can improve learning and memory abilities, promote the degradation of A*β*, and clear amyloid plaques from the AD brain (He, et al., 2013; Liu, et al., 2011; Yang, et al., 2014). Therefore, we wish to extend these observations in early AD intervention, focusing on amyloid deposition and synaptic plasticity in this study.

## Materials and Methods

### Animals

We used 4-month-old APP/PS1 transgenic mice purchased from the Model Animal Research Center of Nanjing University. Transgenic mice were randomly divided into five groups: APP/PS1, Aricept, PNS, GP, and NP groups (*n* = 14). Their littermate wild-type mice were the control (*n* = 14). Mice were kept in cages (two mice per cage) and maintained at a constant temperature on a 12-h light–dark cycle with access to food and water freely. All procedures concerning the care, treatment and dissection are following the Animal Ethics Committee of Beijing University of Chinese Medicine (No. BUCM-4-2016040301-2001).

### Drug Administration

**FIGURE 1:**
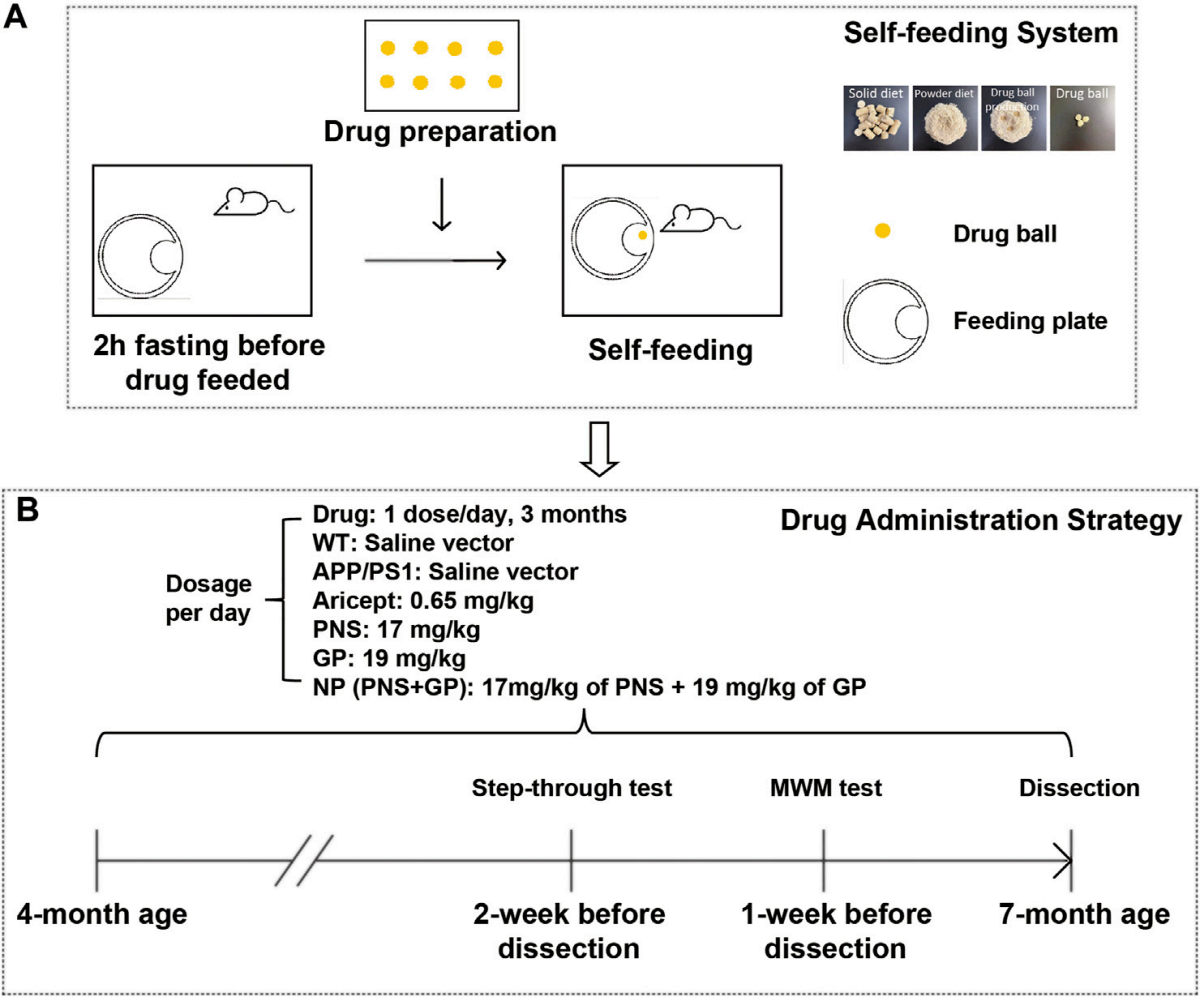
Introduction of the self-feeding system and early intervention strategy. **(A)** Sketch of the self-feeding system. Drugs were mixed in food powder to produce drug balls. Driven by hunger, mice were trained to feed. **(B)** Timeline for early intervention and behavioral experiment schedule in APP/PS1 transgenic mice.

Based on previous studies (Yang, et al., 2014), the dosage of these drugs was converted according to the body weight index between human beings and mice. PNS was 17 mg/kg.d, GP 19 mg/kg.d, and Aricept 0.65 mg/kg.d. NP is the combination of PNS and GP. Drugs were given once a day for 3 months. WT and APP/PS1 groups have equal volume vectors. To minimize the stress effect caused by intragastric administration, we developed a three-step self-feeding system to guide mice to feed (Figure 1): 1) Food ball or drug ball preparation. Food pallets were ground into powder, and food ball was produced by mixing with 200 µL saline or drug solution. 2) Feeding habit management. We trained the mice of feeding habit for 1 week. From 7–9 a.m., we removed food pallets to drive hunger. During that time, we put a food box at the edge of the cage. After 2 hours of fasting, the food ball or drug ball was applied in the food box. Due to hunger, the mice promptly grabbed the food ball and ate it up. One hour later, food supply was provided as normal. 3) Drug administration. After the training, we found that mice ate the food ball within 5 min.

### Golgi Staining

**FIGURE 2:**
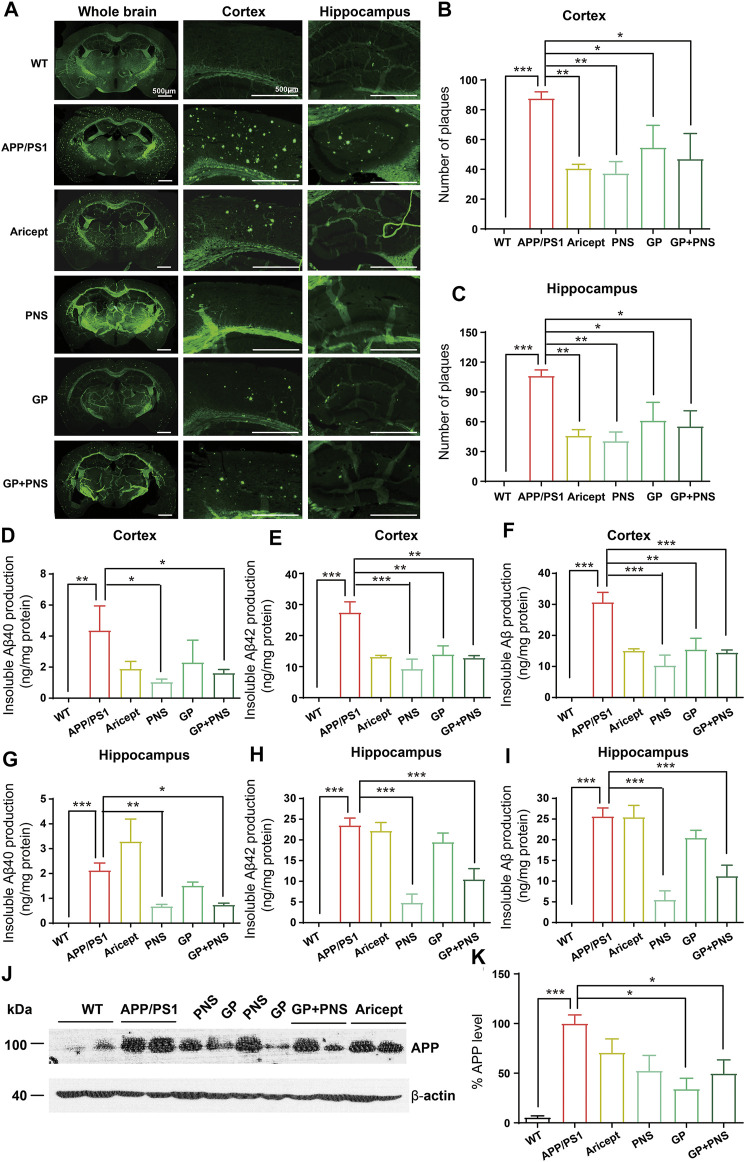
Reduction of Aβ production and amyloid deposits. **(A)** The typical feature of amyloid deposits in brains. **(B)** The number of amyloid plaques was analyzed in the cortex. **(C)** The number of amyloid plaques was analyzed in the hippocampus. **(D-F)** The amounts of insoluble Aβ40 **(D)**, insoluble Aβ42 **(E)** and insoluble Aβ in total **(F)** were analyzed in the cortex. **(G-I)** The amounts of insoluble Aβ40 **(F)**, insoluble Aβ42 **(G)** and insoluble Aβ in total **(I)** were analyzed in the hippocampus. **(J-K)** The Western blot of APP **(J)** and analysis **(K)**. (n = 5, Data are means ± SEM. Compared with APP/PS1, *p < 0.05, **p < 0.01, and ***p < 0.001; one-way ANOVA, followed by Tukey’s multiple comparison test).

Golgi staining was performed using the Hito Golgi-Cox OptimStain^TM^ Kit, according to the manufacturer’s instructions. In brief, the fresh brain was kept in the mixture impregnation solution at room temperature in the dark and replaced into the impregnation solution on the next day. The brain was stored for 2 weeks, following the replacement of solution 3 for 48 h at 4°C and preparing 150-μm tissues sections. Neurons were stained with the mixture of solution 4, 5, and distilled water (1:3:5 ratio) for 10 min. The slides were dehydrated by continuous processes and mounted using a mounting medium. For a dendritic structure analysis, we selected neurons (5 per animal) located in the CA1 region of the hippocampus. These neurons must be visually inspected for the integrity of dendritic branches and isolated from neighboring neurons to avoid interference of synaptic tracing by Image-Pro Plus. The dendritic length from the soma and the number of intersecting radii every 10 μm were used to estimate dendritic arborization complexity (Figures 2A,B). Furthermore, we randomly chose the second dendritic branches at both the basal and apical segments. Images of dendritic spines were taken using the confocal microscope (Olympus; magnification, 100 ×) and calculated.

### Western Blot

Western blot and analysis were carried out, as described previously (Liu et al., 2011). In brief, proteins (5–10 μg of total protein) were separated by SDS-PAGE, transferred to PVDF membranes, and blocked in 5% skimmed milk. The primary antibodies were rabbit anti-APP (1:2000, ab241592, Abcam), rabbit anti-PSD-95 (1:1000, ab238135, Abcam), or mouse anti-*β*-actin (1:4000, #3700, CST). The secondary antibodies were corresponding horseradish peroxidase–conjugated ones. A chemiluminescent substrate kit (Thermo Fisher Scientific, United States) was used and developed by using an X-ray film (Kodak) in the dark room. The developed films were scanned, and the percentage of the band relative intensity was analyzed by ImageJ software.

### Analysis of Deposition of Amyloid Plaques

Brains were dissected and processed for serial paraffin sections (6 µm). By conventional dewaxing and rehydration, sections were immersed for 20 min in 0.1% Thioflavine-S (Sigma-T1892, Sigma-Aldrich, United States) PBS solution at room temperature. The green fluorescence-stained plaques were visualized by fluorescence microscopy. The number of plaques in the cerebral cortex and hippocampus was analyzed respectively.

### ELISA

The hippocampal and cortical homogenates were collected for supernatant fraction and allowed to precipitate. The pellet was further re-suspended in 70% formic acid, received sonication for 10 s, and was centrifuged at 17,000 rpm for 1 h at 4°C. The supernatant was collected for the insoluble A*β* ELISA assay. The insoluble levels of A*β*1-40 and A*β*1-42 were determined by using commercial kits (KHB3481 and KHB3441, Invitrogen, United States), according to the manufacturer’s instructions.

### Morris Water Maze (MWM)

MWM is a classical method to evaluate hippocampal-dependent spatial learning and memory. Before the behavioral test, mice were acclimatized to the laboratory room for at least 30 min. The test took place between 8 a.m. up to 4 p.m. The apparatus consisted of a white circular pool (80 cm in diameter and 50 cm deep) divided into four equal imaginary quadrants for data analysis. Water was mixed with milk powder, and the temperature was maintained between 21 and 23°C. The hidden platform is 10 cm in diameter and placed at one quadrant. Three visual stimuli hung on the curtains that surrounded the pool. Mice underwent two separate trials, a training trial, and a test trial. For the training trial, mice were trained to reach the hidden platform within 60 s; if the mouse failed to find the platform, the experimenter guided it and allowed the mouse to stand on the platform for 10 s. Each mouse had four trials per day for every four quadrants. The training trial was conducted for five consecutive days. For the test trial, on the sixth day, the platform was removed, and the mice were released at the opposite quadrant. The latency to the platform and the number of crossings over the platform area were recorded. Also, the swimming speed was calculated to confirm the standard moving capability.

### Shuttle Box Test (Passive Avoidance Performance)

The passive avoidance shuttle box test is a widely accepted rapid and straightforward method to evaluate fear memory. The apparatus consisted of two same size compartments separated by a wall with a guillotine door. One of the two chambers was illuminated, and the other was dark. Floors constructed of 3.175-mm stainless-steel rods were set 8 mm apart and connected to a shock generator. Mice freely adapted to the apparatus for 5 min After adaption for 24 h, mice underwent two separate trials, a training trial (5 min each mouse) and a test trial (10 min each mouse). For the training trial, mice were initially placed in the illuminated chamber. When mice entered the dark compartment, an electrical foot shock (0.4 mA) of 2 s duration was delivered through the stainless-steel rods. For the test trial, given 1 day after the training trial, the procedure was performed in the same manner without the electric shock and the latency of step through to the dark compartment and the error in 10 min were recorded as the reflection of the learning. The latency to enter the dark compartment recorded up to 600 s.

### Statistical Analysis

All the analyzed data presented as mean ± standard error of the mean (S.E.M.). We performed one-way ANOVA or two-way ANOVA followed by *post hoc* comparison of the mean ± S.E.M. using Tukey’s multiple comparison test and Bonferroni’s or Dunnett’s T3 methods. Values of *p* < 0.05 were considered as statistically significant. The graphical abstract was created with BioRender.com.

## Results

### Introduction of the Self-Feeding System and Early Intervention Strategy

To mimic self-feeding without stress, we designed a self-feeding system to guide mice to take drugs (Figure 1A). First, mice accommodated the feeding system for 1 week. Two days before drug feeding, mice fasted 2 h earlier than the food balls provided. Driven by hunger, they took the food ball spontaneously. One hour later, we provided the normal food supply. After the training, 4-month-old APP/PS1 transgenic mice were administered with the drug and continued for 3 months. The timeline for early intervention and the schedule for behavioral experiments are shown in Figure 1B.

### Reduction of A*β* Production and Amyloid Deposits

To assess A*β* pathology in the brains, we observed amyloid deposits and insoluble A*β* production in the cortex and hippocampus. First, we illustrated the typical feature of the whole brain by Thioflavine-S staining (Figure 2A). No positive staining was presented in WT mice, but we observed numerous amyloid plaques in the brains of APP/PS1 transgenic mice. The number of amyloid plaques in all intervention groups decreased significantly (Figures 2B,C).

Accordingly, no insoluble A*β* peptide was detected in WT mice. In contrast, APP/PS1 mice showed significantly higher levels of insoluble A*β* peptides (Figures 2D–I). In addition, NP and PNS significantly decreased the levels of A*β*40 and A*β*42 in both cortex and hippocampus areas (Figures 2D–I). GP only showed the effect on the production of A*β*42 in the cortex (Figure 2E). Furthermore, we examined the amyloid precursor protein (APP) protein level in the cortex. We found that PNS did not change the protein expression of APP, while GP significantly reduced the APP expression (Figures 2J,K).

### Enhancement of the Synaptic Plasticity

**FIGURE 3:**
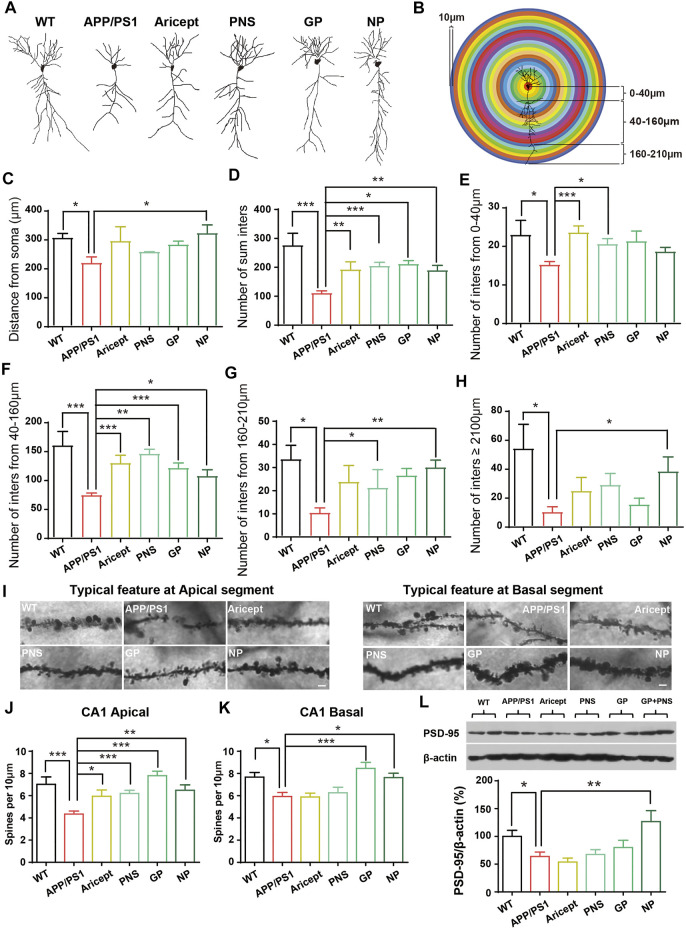
Enhancement of the synaptic plasticity. **(A)** Typical feature of neurons by tracing analysis. **(B)** Experimental model for dendritic structure analysis. The number of intersections was calculated per 10 µm. **(C)** Distance from soma. **(D)** Number of intersections in total. **(E–H)** Number of intersections in the range of 0–40 µm, 40–160 µm, 160–210 µm, and more than 210 µm. **(I)** Typical feature of spines at CA1 apical and basal segments. **(J–K)** Number of spines at CA1 apical and basal segments was analyzed. **(L)** Expression of PSD-95 protein in the hippocampus. (*n* = 5, Data are means ± SEM. Compared with APP/PS1, *p < 0.05, **p < 0.01, and ***p < 0.001; one-way ANOVA, followed by Tukey’s multiple comparison test).

We next observed the dendritic structure since their enrichment is impaired early than clinical pathologies and degenerated throughout AD processing. The typical structure of CA1 pyramidal cells is shown in Figure 3A. Since the length of dendrites and their branches seem enriched under drug administration, we further measured the distance of dendrites from soma and the number of intersections per 10 µm from soma to 0–40 μm, 40–160 μm, 160–210 μm, and more than 210 µm accordingly (Figure 3B). Analyzing neuron tracing, APP/PS1 mice showed the shorter length of dendrites, and only NP significantly promoted the length (Figure 3C). Then, the number of intersections per 10 µm was analyzed accordingly since the intersection number considered another sign of dendritic structure richness. The number of intersections was analyzed in four categories: 0–40 μm, 40–160 μm, 160–210 μm, and more than 210 µm. In terms of the total number of intersections, all the intervention groups were significantly higher than APP/PS1 mice (Figure 3D). By category, the positive drug Aricept showed significant richness at the near distance, 0–160 μm, while NP showed significant richness at the far distance from the soma, 40–210 µm and ≥210 µm (Figures 3F–H). PNS or GP partially contributed to the richness of intersection (Figures 3E–G). In addition, Golgi staining calculated the apical and basal spinal density. All the intervention groups significantly increased the spine number at the apical spinal density of the pyramidal cells. Only NP and GP significantly increased the basal spine density (Figures 3I–K).

Furthermore, we detected the synaptic function-related protein level by using Western blot. APP/PS1 mice had a lower protein level of PSD-95 compared with their littermate WT control. Only NP significantly increased the expression of PSD-95 in the hippocampus (Figure 3L). These results indicated that NP enriched the dendritic structure and promoted synaptic-related protein expression in the hippocampus.

### Improvement of Learning and Memory Performance

**FIGURE 4:**
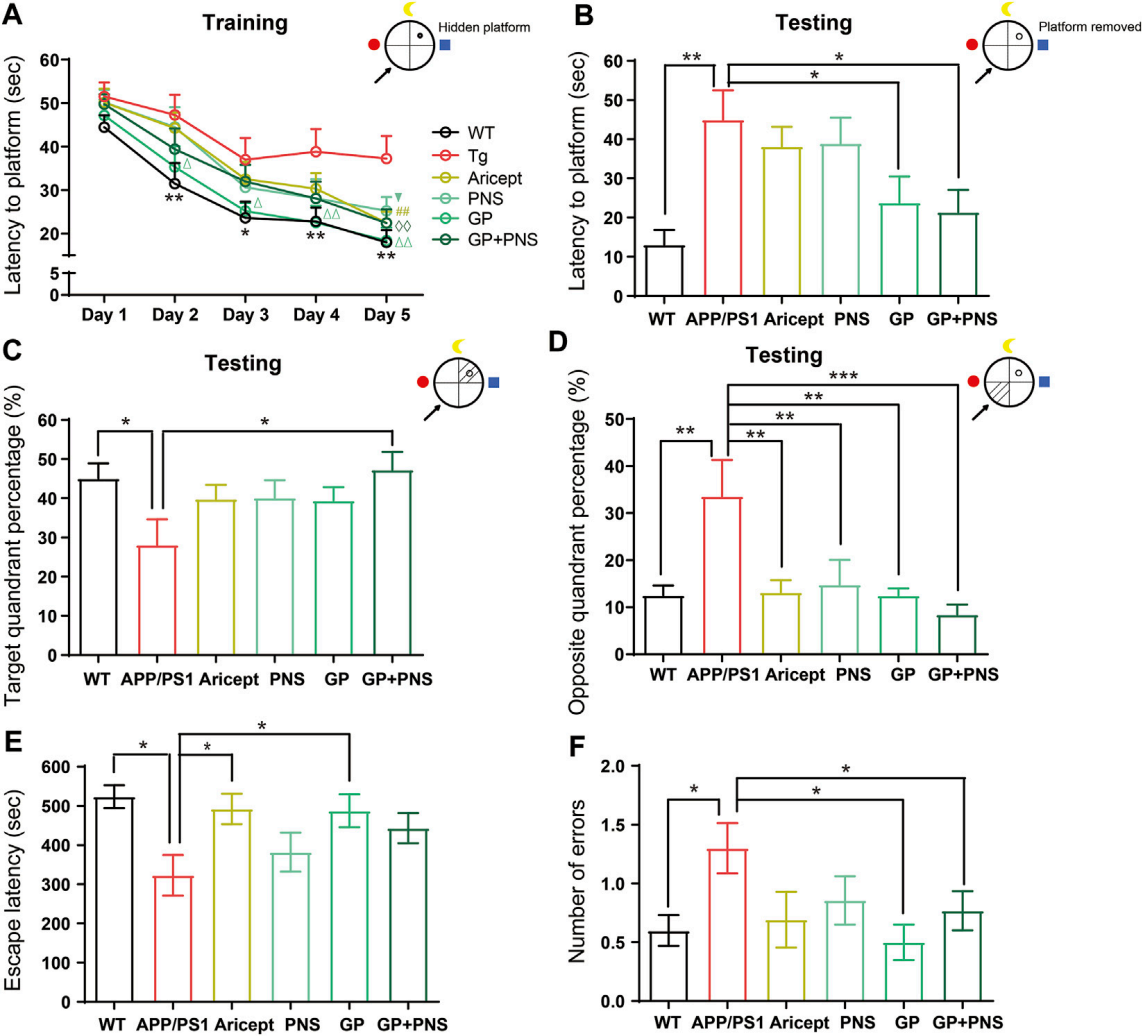
Learning and memory performance enhancement. **(A)** In the training trial of MWM, the latency to platform. **(B)** In the testing trial of MWM, the latency to platform. **(C,D)** In the testing trial of MWM, the percentage of target quadrant **(C)** and opposite quadrant **(D)**. **(E,F)** In the avoidance test, the escape latency from electric shock **(E)** and the error number to the electric shock area **(F)**. (*n* = 12, Data are means ± SEM. Compared between WT and APP/PS1, **p < 0.01 and *p < 0.05, compared between NP and APP/PS1, ◊◊*p* < 0.01; compared between GP and APP/PS1, ∆∆*p* < 0.01 and ∆*p* < 0.05; compared with PNS and APP/PS1, ▼*p* < 0.05; compared between Aricept and APP/PS1, ##*p* < 0.01 (two-way ANOVA for **(A)**. One-way ANOVA for others, compared with APP/PS1, ***p < 0.001, **p < 0.01, and *p < 0.05.).

We next confirmed the effectiveness of using behavioral tests. Using the MWM test, the escape latencies assessed the ability of mice to acquire and recall spatial information. In the training trial, the mean escape latency was recorded from four quadrants in successive 5 days. The results showed a significant difference between APP/PS1 mice and their WT littermates (Figure 4A, *p <* 0*.*01), and the differences among GP mice and transgenic mice were also significant (Figure 4A, *p <* 0*.*05) from the second day to the fifth day. On the final training day, Aricept- or PNS- or NP-treated mice showed significant differences compared with transgenic mice (Figure 4A, *p <* 0*.*01, *p <* 0*.*05, *p < 0.0*1). In the testing trial, removal of the platform, APP/PS1 mice showed a significantly longer time to reach the platform area (Figure 4B, *p* < 0.01). Only NP significantly shortened the latency or the percentage of time on the target quadrant compared with APP/PS1 mice (Figures 4B,C, *p* < 0.05 C), while all the intervention groups significantly shortened the duration at the opposite quadrant (Figure 4D).

The passive avoidance test was also applied to further confirm the fear memory. Compared with WT mice, APP/PS1 mice showed a significantly shorter step-through time. NP, Aricept, and GP significantly increased the duration compared with the APP/PS1 group (Figure 4E). In addition, APP/PS1 mice significantly increased the number of passing errors, while GP and NP significantly reduced the error number (Figure 4F). Together, NP can significantly improve spatial learning and memory and fear memory in the 7-month-old APP/PS1 mice. The spatial learning seemed partially contributed by both PNS and GP, while the fear memory was mainly contributed by GP.

## Discussion

AD is the most common cause of dementia. Based on a biological definition of AD, the prevalence of dementia will double in Europe and triple worldwide by 2050 (Scheltens, et al., 2021). In China, dementia, especially AD, is quickly increasing. As reported, the prevalence of AD was 3.21% in people aged more than 65 years (Jia, et al., 2014). Accompanied by the high prevalence, AD is becoming one of the most burdening diseases for the elderly. The global estimates of costs for dementia will be $2.54 trillion in 2030 and $9.12 trillion in 2050 (Jia, et al., 2018). However, what is not compatible with the situation is that AD’s diagnosis and treatment rate are low; more than half of individuals living with AD are not getting an accurate diagnosis (Burke and Apostolova, 2021). Currently, drugs for AD are mainly developed for mild to moderate AD (Dhillon, 2021; Ketter, et al., 2017; Salloway, et al., 2014), but neurodegeneration occurs earlier than the clinical diagnosis (Ausó, et al., 2020; Ingelsson, et al., 2004). In early 2018, the FDA has proposed that efforts are particularly significant to intervene very early in AD processing (U.S. Department of Health and Services, 2018). TCM has the same concept of preventing diseases. A systematic review also addressed that Chinese herbal medicine (CHM) alone or as an adjuvant treatment showed significant effects in improving cognitive functions of AD (Liu, et al., 2021).

In our team, previously, we found that the combination of *Panax notoginseng* and *Gardenia jasminoides* Ellis can improve cognitive functions in acute stroke patients (Chinese SFDA: 2004 L01620). We proved that the combination improved the learning and memory abilities in three different AD-like animal models (He, et al., 2013; Liu, et al., 2011; Yang, et al., 2014). Based on these observations, we aimed to expand the application of NP in prevention. APP/PS1 transgenic mice expressed a human amyloid precursor protein (HuAPP695swe) and a mutant human presenilin 1 (PS1-dE9), both associated with early-onset AD. The drug administration strategy started in 4-month-old APP/PS1 mice since A*β* started to accumulate; drug administration lasted for 3 months; at the age, amyloid deposition was apparent and memory deficits occurred (Guo, et al., 2015; Jankowsky, et al., 2004).

Our results confirmed that NP significantly reduced the number of amyloid depositions and decreased the insoluble A*β* production (Figures 2A–G). NP significantly reduced the protein level of APP. A*β* is generated from the cleavage of APP by two enzymes: *β*-secretase (BACE1) and γ-secretase. Emerging evidence has shown that BACE1 expression levels and activities were increased in the brain of AD patients (Ahmed, et al., 2010; Hampel, et al., 2021). However, the levels and activities of *γ*-secretase remain controversial (Veugelen, et al., 2016; Xia, et al., 2016; Xia, et al., 2015). Several enzymes, such as neprilysin (NEP) and insulin-degrading enzyme (IDE), were further involved in A*β* degradation (Jha, et al., 2015; Wang, et al., 2010). Previously, we found that NP (also called TLJN in previous studies) downregulated the levels and activity of BACE1 and decreased the protein levels of PS1, nicastrin, and APH1, indicating to effect on APP processing (He, et al., 2013). PNS is a mixture of R1, Rd, Rb1, Re, and Rg1 extracted from *Panax notoginseng* (*San Qi*). Related studies proved that Re and Rg1 significantly reduced A*β* production by inhibiting the BACE1 activity (Cao, et al., 2016; Tan, et al., 2022). Also, NP increased NEP and IDE protein levels, indicating promotion of the degradation of A*β* (Liu, et al., 2011). In addition, GP extracted from *Gardenia jasminoides* Ellis also showed neuroprotective effects on AD pathology (Dinda, et al., 2019; Zhao, et al., 2017). GP accelerated APP degradation by increasing the activity of unfolded protein responses (Cui, et al., 2018). Together, we addressed that NP significantly reduced the amyloid deposits by modulating APP processing and A*β* degradation. PNS may highly work downstream of APP processing, while GP affected the APP protein level.

The positive drug Aricept is one of the FDA-approved drugs for AD therapy, acting as an acetylcholinesterase (Ach-E) inhibitor (Anand and Singh, 2013). Animal studies found that Aricept reduced the levels of soluble and insoluble A*β* and amyloid plaques in the hippocampus by using 3 × Tg AD mice (APP_Swe_/PSEN_M146V_/MAPT_P310L_) (Zhou, et al., 2019). This seemed inconsistent with our observation. From our data, we noticed that Aricept decreased the number of amyloid plaques in the cortex and hippocampus areas, but it cannot affect the production of insoluble A*β* in the hippocampus. However, the variation can happen because of different animal models and drug administration strategies. We also found that studies addressed that Aricept inhibited A*β*1-42 self-aggregation and Ach-E-induced A*β*1-40 aggregation (Mezeiova et al., 2019a); it enhanced A*β* clearance across the blood–brain barrier (Mohamed, et al., 2015). These indicated that Aricept plays a key role in inhibiting A*β* aggregation rather than its production (Mezeiova et al., 2019b). However, long-term Aricept treatment was associated with more significant amyloid load and tau burden in the temporal lobe, posterior cingulate, parahippocampal gyrus, and worse cognitive performance among individuals with mild cognitive impairment (Li, et al., 2022). One year of Aricept administration did not reduce brain A*β* accumulation in human patients with AD (Ishibashi, et al., 2017).

AD also refers to a progressive and synaptic failure disease. It is well documented that the loss of synaptic contacts occurred in both the neocortex and hippocampus. Synaptic density demonstrated a strong association with cognitive performance (Scheff, et al., 2006); this correlation can extend to the early stages of AD (Mecca, et al., 2022). The synapse is where A*β* peptides are generated and is the target of the toxic A*β* oligomers (Pelucchi, et al., 2022). In particular, emerging data suggest that cognitive decline would occur only in the setting of A*β* accumulation with synaptic dysfunction (Sperling, et al., 2011). Then, identifying and treating these early markers altogether are the hopes of investing in this field of study (John and Reddy, 2021). In order to illustrate the potential role in AD processing, we also focused on synaptic plasticity. Research found that PNS ameliorated synaptic dysfunctions by suppressing overactivation of NMDA receptors (Yan, et al., 2014; ZhouYi-Jun et al., 2020). GP alleviated neuroinflammation by inhibiting HMGB-1 and downregulating TLR4/2 and RAGE signaling pathways *in vivo* (Lv, et al., 2015; Zhou Zhangjiuzhi et al., 2020). GP-activated GLP-1 receptor, an essential kinase regulating energy balance, proliferation, and survival in cells, reduces amyloid plaques and inhibits synaptic loss (Zhang, et al., 2021; Zhang, et al., 2019). From our data, GP exhibited relatively more minor effects on synaptic branch richness but performed better at the spine density of CA1 pyramidal neurons (Figures 3J,K). Through behavioral tests, both PNS and GP partially contributed to memory enhancement, but GP mainly contributed to the fear memory (Figures 4E,F). Together, PNS and GP partially contributed to neuroprotection which makes the combination effects more obvious (Tables 1, 2).

**TABLE 1:** Effects on the APP processing and synaptic structure.

Testing/Grouping	APP processing			Dendritic and synaptic structure					
	Plaque deposit	A*β* production	APP expression	Distance from soma	Number of intersections		Spine density		PSD-95 expression
					Near to soma (0–160 μm)	Far from soma (more than 160 μm)	CA1 apical	CA1 basal	
APP/PS1^a^	↑	↑	↑	↓	↓	↓	↓	↓	↓
Aricept^b^	↓	↓ only cortex	N.S.	N.S.	↑	N.S.	↑	N.S.	N.S.
PNS^b^	↓	↓	N.S.	N.S.	↑	↑	↑	N.S.	N.S.
GP^b^	↓	↓ only cortex	↓	N.S.	↑	N.S.	↑	↑	N.S.
NP^b^	↓	↓	↓	↑	↑	↑	↑	↑	↑

^a^Compared with WT.

b Compared with APP/PS1.

**TABLE 2:** Effects on the behavioral tests.

Testing/Grouping	Behavior tests		
	Morris water maze		Step-through test
	Spatial learning	Spatial memory	Fear memory
APP/PS1^a^	↓	↓	↓
Aricept^b^	↑	N.S.	↑
PNS^b^	↑	N.S.	N.S.
GP^b^	↑	N.S.	↑
NP^b^	↑	↑	↑

a Compared with WT.

b Compared with APP/PS1.

In conclusion, our findings suggested that NP supplemented into food attenuates A*β* pathology, promotes synaptic plasticity, and enhances memory function in the AD animal model. As noted, the administration started as the A*β* pathology began to appear. NP might be useful for AD prevention. In the future, NP could develop as a health food supplement, or its raw materials could be used as medicine to prevent or treat AD. This study contributed to developing a candidate TCM formula for the treatment of AD.

## Data Availability

The raw data supporting the conclusion of this article will be made available by the authors, without undue reservation.
